# Neonatal Thromboembolic Disease: Clinical Spectrum and Emerging Evidence for Diagnostic and Therapeutic Strategies Addressing Unmet Needs

**DOI:** 10.3390/jcm15166221

**Published:** 2026-08-11

**Authors:** Rozeta Sokou, Alexandra Lianou, Vasiliki Mougiou, Andreas G. Tsantes, Stefanos Bonovas, Argirios E. Tsantes, Nicoletta Iacovidou

**Affiliations:** 1 Neonatal Department, Aretaieio Hospital, National and Kapodistrian University of Athens, 11528 Athens, Greece; niciac58@gmail.com; 2 Neonatal Intensive Care Unit, “Aghios Panteleimon” General Hospital of Nikea, 18454 Piraeus, Greece; alexlianou@med.uoa.gr; 3 Neonatal Unit, Royal Victoria Infirmary, Newcastle upon Tyne NE1 4LP, UK; vassouli@hotmail.com; 4 Laboratory of Haematology and Blood Bank Unit, “Attikon” Hospital, School of Medicine, National and Kapodistrian University of Athens, 11528 Athens, Greece; andreas.tsantes@yahoo.com (A.G.T.); atsantes@yahoo.com (A.E.T.); 5 Department of Biomedical Sciences, Humanitas University, 20090 Pieve Emanuele, Italy; 6 IRCCS Humanitas Research Hospital, 20089 Rozzano, Italy

**Keywords:** neonatal thromboembolic disease, thrombosis, catheter-related thrombosis, neonatal hemostasis, anticoagulant treatment

## Abstract

Neonatal thromboembolic (TE) disease is an increasingly recognized clinical entity, driven by the physiological uniqueness of developmental hemostasis, improved survival of preterm infants, and the widespread use of invasive supportive technologies. Most neonatal thrombotic events are associated with central venous and arterial catheterization which are considered the most significant modifiable risk factors. The clinical spectrum encompasses catheter-related thrombosis, perinatal stroke and site-specific entities such as renal vein and portal vein thrombosis. Each condition presents distinct challenges such as neurological complications, chronic organ dysfunction and portal hypertension. Diagnosis relies primarily on Doppler ultrasonography, though its sensitivity is limited in deep-vessel scenarios, often requiring advanced modalities like magnetic resonance imaging (MRI) or magnetic resonance angiography (MRA). Therapeutic interventions remain controversial due to a lack of high-quality evidence, with current guidelines largely based on observational data. Ultimately, managing neonatal TE requires a nuanced, individualized approach that balances the risk of thrombus extension against the inherent hemorrhagic vulnerability of the neonate. This review aims to provide a comprehensive and up-to-date overview of neonatal TE, emphasizing major clinical entities and diagnostic strategies. It highlights current evidence gaps and outlines future directions, including the need for standardized management protocols and high-quality prospective research.

## 1. Introduction

Neonatal thromboembolic (TE) disease represents a complex and increasingly recognized clinical entity that reflects both advances in neonatal care and the unique physiological characteristics of haemostasis during early life [1,2,3]. Over recent decades, the reported incidence of neonatal thrombosis has risen. This apparent increase appears to reflect primarily the improved survival of extremely preterm and critically ill neonates, the widespread use of central vascular access and other invasive supportive interventions, as well as the enhanced sensitivity of modern imaging modalities, rather than a true increase in disease incidence [4]. Despite these advances, neonatal TE remains challenging, characterized by heterogeneous clinical presentations, variable outcomes, and a limited high-quality evidence base to guide management [5,6,7].

Physiological differences in coagulation factors, anticoagulant proteins, and fibrinolytic pathways distinguish neonates from older children and adults, and influence both thrombotic risk and response to therapy [8]. These age-specific characteristics complicate diagnostic evaluation and necessitate a tailored clinical approach. Most neonatal thrombotic events are provoked and occur in the context of identifiable risk factors. Central venous and arterial catheter use represents the most significant contributor. Additional predisposing conditions include prematurity, perinatal asphyxia, sepsis, congenital heart disease, and maternal or perinatal complications [3,9,10,11,12], as shown in Table 1. Clinical manifestations of thrombosis in this population range from asymptomatic, incidentally detected events to life-threatening complications affecting vital organs such as the brain, kidneys, and gastrointestinal tract [3,13,14,15].

The spectrum of neonatal TE encompasses catheter-related thrombosis, perinatal stroke-including arterial ischemic stroke (AIS), and cerebral sinovenous thrombosis (CSVT)-as well as site-specific entities such as renal vein and portal vein thrombosis [5,6,7]. Each condition carries distinct pathophysiological mechanisms, clinical implications, and management challenges. Notably, perinatal stroke represents a leading cause of long-term neurological morbidity, while renal and portal vein thromboses may result in chronic organ dysfunction and delayed complications such as hypertension or portal hypertension [1,13,15,16,17]. Diagnosis relies primarily on imaging modalities, with Doppler ultrasonography serving as the cornerstone in most clinical scenarios, although its diagnostic accuracy may be limited by technical and patient-related factors, further complicating clinical decision-making [14,18,19].

This narrative review aims to provide a comprehensive and up-to-date overview of neonatal thromboembolic disease. It focuses on major clinical entities, as well as diagnostic and management strategies, while highlighting existing knowledge gaps and future research priorities.

This review was based on a comprehensive literature search conducted in the PubMed/MEDLINE, Scopus, and Google Scholar databases, focusing on studies published up to June 2026. The search strategy employed combinations of keywords including “neonatal thrombosis,” “neonatal thromboembolism,” “venous thromboembolism,” “arterial thrombosis,” “cerebral sinovenous thrombosis,” “renal vein thrombosis,” “portal vein thrombosis,” “catheter-related thrombosis,” “developmental hemostasis,” “anticoagulation,” “thrombolysis,” “newborn,” and “neonate,” together with terms related to diagnosis, treatment, and clinical outcomes. Priority was given to the most recent international clinical practice guidelines (ASH and ISTH), systematic reviews, meta-analyses, randomized controlled trials where available, as well as large observational studies and neonatal cohort studies. Additional relevant publications were identified through manual screening of the reference lists of the selected articles.

**Table 1:** Incidence and main etiological risk factors associated with neonatal thromboembolism.

Risk Factor/Cause	Incidence Range Across Included Cohorts	References
Central Vascular Access Devices (CVL/CAD/UVC)	35.7–75.0%	[3,11,12,20]
Sepsis and Bloodstream Infection	14.3–69.4%	[3,11,12,20]
Prematurity (<37 Weeks Gestation)	23.0–58.3%	[3,11,20]
Mechanical Ventilation/Respiratory Support	22.0–73.7%	[3,11,20]
Maternal Risk Factors (DM, Hypertension, PROM, Preeclampsia)	10.7–36.1%	[11,12,20]
Inherited Thrombophilia (Genetic Mutations)	14.2–69.0%	[11,12]
Congenital Heart Disease	8.8–32.0%	[3,11,12]
Perinatal Asphyxia/Hypoxic–Ischemic Encephalopathy	5.3–6.4%	[20]

Summary ranges of predisposing clinical conditions, catheter interventions, and thrombophilic risk factors reported across major neonatal cohorts. CVL, central venous line, CAD, central access device, UVC, umbilical vein catheter, DM, diabetes mellitus, PROM, premature rupture of membranes.

## 2. Neonatal TE Associated with the Presence of Central Catheters

**Figure 1:**
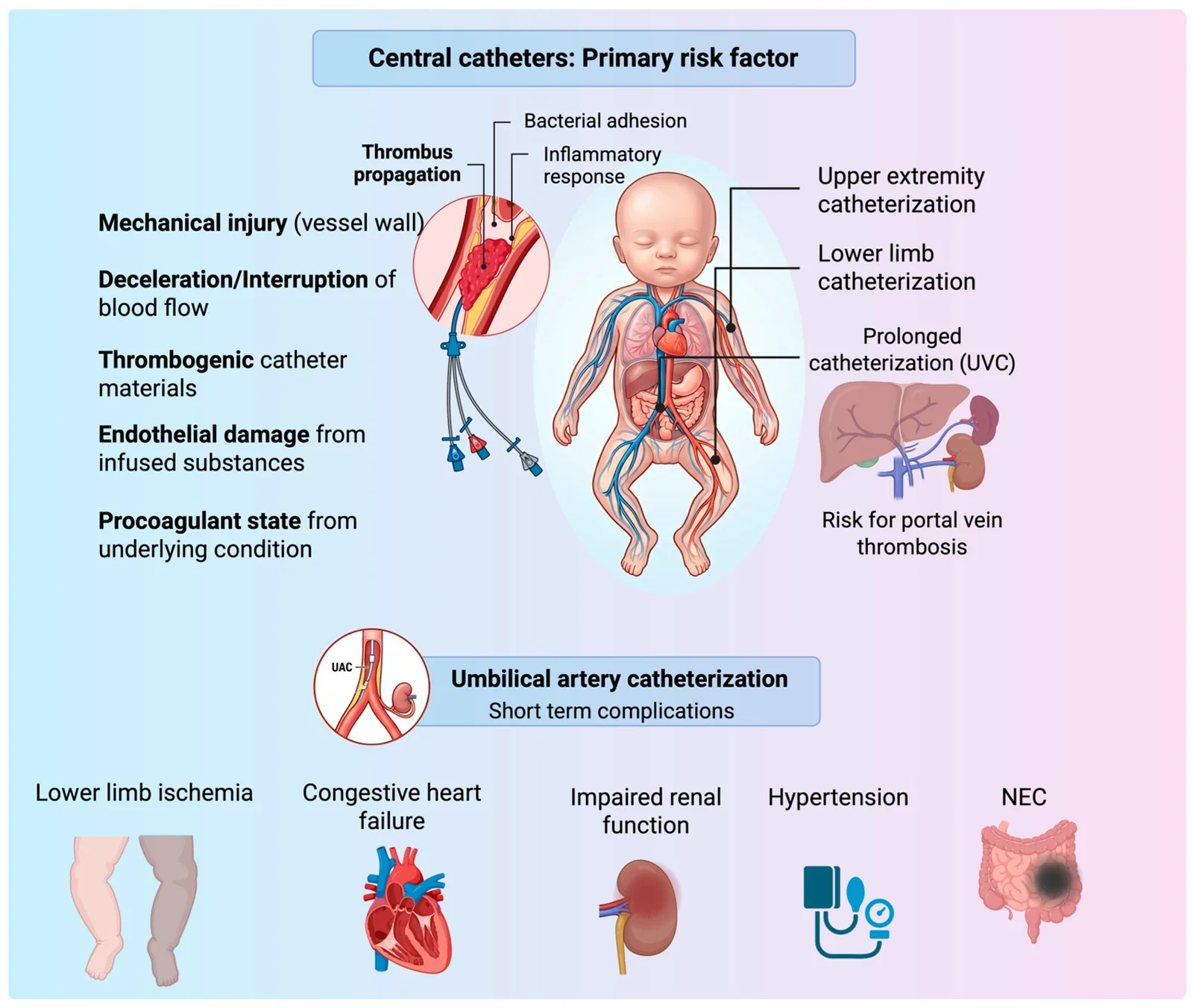
**Risk factors for neonatal thromboembolism.** Neonatal thromboembolism (TE) is strongly associated with central and arterial catheters, which are major risk factors due to their potential to damage vessel walls, disrupt blood flow, and promote clot formation. Although improved care has reduced catheter-related events, they remain a leading cause of both venous and arterial thrombosis, with risks influenced by catheter type, placement, and duration [5]. Venous thrombosis is commonly linked to CVC and may require anticoagulation, while arterial thrombosis—often related to umbilical or peripheral arterial catheters—can lead to serious short- and long-term complications. UVC: umbilical vein catheter, NEC: necrotic enterocolitis [29,30]. Created in BioRender. Lianou, A. (2026) https://BioRender.com/rv9rpka.

The apparent increase in thrombotic disease in neonates recorded over the years can be attributed to the higher survival rates of extremely preterm infants, the increased use of central venous catheters (CVC), and the higher sensitivity of imaging modalities to diagnose thrombosis. Central catheterization is indisputably the most important risk factor for both arterial and venous thrombosis [5,6,7]. The presence of a CVC promotes the development of thrombotic events with various mechanisms, either by itself or in combination with other factors. To be specific, the catheter may cause mechanical injury to the vascular wall and deceleration or interruption of blood flow. In addition, the materials used in catheter construction may be thrombogenic, while substances infused through catheters may damage the vascular endothelium. In addition, underlying comorbidities that require CVC placement often lead to a procoagulant state [5]. The presence of a catheter often results in the formation of a fibrin sheath around it, which promotes bacterial adhesion and subsequent bacterial growth and this, in turn, leads to an inflammatory response and thrombus propagation [14,21] (Figure 1). Moreover, central line malposition in neonates is an uncommon but potentially serious cause of morbidity. Catheter malposition contributes to thrombogenesis through multiple mechanisms. An improperly positioned catheter tip causes repeated mechanical trauma to the vascular endothelium, alters normal blood flow with the development of turbulence and stasis, and may expose the vessel wall to hyperosmolar infusates. These processes promote endothelial dysfunction, activation of the coagulation cascade, fibrin sheath formation, and ultimately thrombus development [22,23]. Sepsis and the presence of a CVC are the two major contributors to neonatal thrombosis. The catheter provides a thrombogenic surface that promotes coagulation activation, while catheter-related infection further enhances endothelial injury and activation of the coagulation cascade, thereby increasing the risk of thrombus formation, particularly during prolonged catheterization [24]. Catheter material has also been proposed as a potential determinant of thrombosis risk. Adult studies, particularly in patients with hemodialysis catheters, suggest that catheter materials differ in their thrombogenicity and biocompatibility, with silicone generally demonstrating lower thrombogenic potential than polyurethane [25,26,27]. Comparable evidence in neonatal central vascular catheters remains scarce and inconclusive, and current data do not support preferential selection of a specific catheter material solely for thrombosis prevention. Instead, appropriate catheter tip positioning, minimization of catheter dwell time, and meticulous catheter care remain the cornerstone of thrombosis prevention. Overall adverse event rates have been estimated at 13.4% for umbilical venous catheters and 9% for umbilical arterial catheters. Among these complications, catheter-associated vascular thrombosis occurs in approximately 1–3% of neonates with umbilical arterial catheters. Catheter-related thrombosis remains the most common form of neonatal thromboembolic disease. A recent systematic review and meta-analysis reported pooled incidences of catheter-associated thrombosis of 6.5% (95% CI: 2.28–12.61) among neonates with umbilical venous catheters (UVCs) and 8.2% (95% CI: 1.91–18.20) among those with umbilical arterial catheters (UACs), emphasizing that thrombosis represents one of the major complications of umbilical vascular access [28].

### 2.1. Venous Thrombosis

Several characteristics of CVCs are associated with an increased risk of thrombosis in neonates, including catheter site, the use of multilumen catheters, and prolonged time of catheterisation. A systematic review of the literature demonstrated that prolonged catheterization (>6 days), malposition of umbilical catheters, and administration of blood products via the umbilical catheter were significant risk factors for CVC-associated thrombosis [19,31].

Historically, 89% of thrombotic episodes were attributed to CVCs [7,32], but more recent data indicate that only 54% of venous and 27% of arterial thrombotic events are associated with the presence of a CVC [14]. This reduction in CVC-associated thrombosis is likely attributed to improvements in central line care. A retrospective study from the PHIS database [33], which included 201,033 neonates (2720 of which had a diagnosis of Venus thromboembolism (VTE)) admitted to level III and IV NICUs, demonstrated a significant association between the presence of a CVC and the development of VTE. In this study, CVCs placed in the upper extremities were associated with a higher likelihood of VTE compared with those in the lower extremity, while the simultaneous presence of CVCs in upper and lower extremities was linked with the highest risk of VTE.

The presence of an umbilical venous catheter (UVC) is associated with increased risk of thrombosis, including that of the portal vein. Most catheter-related venous thromboses are clinically silent and are detected incidentally during imaging performed for catheter assessment or unrelated indications. When symptomatic, clinicians should suspect thrombosis in the presence of unexplained catheter dysfunction, difficulty aspirating blood, persistent catheter-related bloodstream infection, limb or neck swelling, superior vena cava syndrome, or unexplained cardiorespiratory deterioration. Doppler ultrasonography represents the first-line imaging modality, whereas echocardiography is essential for intracardiac thrombi and cross-sectional imaging may be required in selected cases. Laboratory findings are nonspecific and mainly reflect the underlying disease rather than the thrombotic event itself [19,31,33]. Diagnosis is typically established using Doppler ultrasonography of the suspected region. For neonates with functional CVC and CVC-associated thrombosis, therapeutic anticoagulation is recommended for 6 to 12 weeks. If the CVC remains in situ, prophylactic anticoagulation is advised until catheter removal, particularly in the presence of ongoing prothrombotic risk factors [34,35]. Previous publications suggested that delaying removal of the CVC for 3 to 5 days after initiation of anticoagulant treatment reduces the risk of embolic events during catheter removal—especially in children with a right-to-left cardiac shunt or those at risk of developing such a shunt [36]. Evidence supporting the optimal timing of central catheter removal remains lacking, and this recommendation is mainly based on empirical observations, expert opinion, and fundamental pathophysiological principles. More recently, the ASH committee noted that practical considerations and resource availability often determine the timing of catheter removal [37]. In cases where thrombosis progresses or embolizes despite sufficient anticoagulation, the decision to remove or maintain a functional CVC should be individualized, taking into consideration venous access availability, the need for ongoing therapy, the risk posed by the underlying condition in which catheter loss may risk treatment continuity, and the availability of a surgeon in case catheter removal is required. The certainty of evidence regarding the impact of central catheter removal on other outcomes remains very low, but the absence of evidence does not imply absence of such an impact [35,37].

### 2.2. Arterial Thrombosis

Nearly all cases of arterial thrombosis in neonates are associated with the presence of arterial catheters [29,30]. Umbilical arterial catheters (UACs) and peripheral arterial catheters (radial, posterior tibial) are commonly used for monitoring of arterial blood pressure and blood gases, while the femoral artery is frequently used for cardiac catheterization. The incidence of arterial catheter–related thrombosis in neonates varies and depends partly on the detection method employed. Specifically, the reported incidence of arterial thrombosis in neonates with a UAC ranges from approximately 8% to 20% [28,29,38]. In a systematic review, the overall incidence of catheter-related arterial thrombosis in neonates with arterial catheters was 21% [30]. Catheter-related arterial thrombotic episodes are most commonly diagnosed following UAC and femoral artery catheterization. Umbilical arterial catheter–related thrombosis (UACRT) is typically asymptomatic and is associated with catheter insertion technique and duration of catheter dwell time [39]. Complications of UACRT may occur, with short-term complications including lower limb ischemia, congestive heart failure, impaired renal function, and hypertension, while necrotizing enterocolitis was reported as a complication following embolization of the superior mesenteric artery [36]. Long-term complications include renovascular hypertension and abnormalities in lower limb growth [36]. Renal failure, necrotizing enterocolitis, and spinal cord infarction are rare complications of UAC-associated thrombosis. These complications occur when the thrombus extends into the renal arteries, mesenteric arteries, or branches of the spinal arteries [18,40,41,42,43,44]. High positioning of the UAC is associated with fewer clinical complications; prophylactic continuous low-dose heparin infusion (1 U/mL) prolongs catheter patency but does not reduce the risk of thrombosis [18,43,44]. Femoral artery thrombosis frequently follows cardiac catheterization procedures. Clinical signs may include features of acute limb ischemia (e.g., pale or discoloured limb) and/or arterial hypertension [30]. Clinical manifestations of peripheral arterial thrombosis include reduced or absent peripheral pulses, reduced perfusion of the limb with prolonged capillary refill time, and cool, pale extremity. Severe thrombosis of a limb may result in long-term arterial insufficiency, affecting the growth of the limb [45]. Arterial thrombosis should be suspected in neonates presenting with diminished or absent peripheral pulses, pallor, cool extremities, prolonged capillary refill, limb cyanosis, or acute hypertension following arterial catheterization. Doppler ultrasonography remains the initial imaging modality, whereas angiographic techniques may be reserved for selected cases requiring detailed vascular assessment. Laboratory investigations are supportive but are not diagnostic [18,40,41,42,43,44,46].

Prophylactic infusion of heparin through arterial catheters may reduce the risk of thrombosis; however, it is not routinely recommended in neonates or children, and the need for it should be evaluated based on each patient’s individual thrombotic risk, as defined by current guidelines [47]. The indication for long-term prophylactic anticoagulation should be individualised, balancing the degree of thrombotic risk (e.g., prior thrombotic events, previous catheter-related thrombosis) with the potential risks of therapy (like bleeding, thrombocytopenia, or heparin-induced osteoporosis). This strategy remains controversial until further studies are carried out. In neonates with a UAC, when prophylactic therapy is employed, it is recommended to administer a low-dose UFH via the catheter. A heparin solution with a concentration of 0.25–1 unit/mL is suggested, with a total daily dose of 25–200 units/kg, aiming at maintaining catheter patency [36].

In a recent multinational survey by the Paediatric and Neonatal Thrombosis and Haemostasis Subcommittee of the Scientific and Standardization Committee (SSC) of the International Society on Thrombosis and Haemostasis (ISTH), current practices and perspectives of paediatric haematologists regarding the management of catheter-related arterial thrombosis in children and neonates were investigated [48]. The findings revealed significant heterogeneity in the duration of antithrombotic therapy, with answers about treatment lengths ranging from 2 weeks to 3 months, showing a similar distribution for UAC, peripheral arterial catheters, and femoral catheters used for cardiac catheterization. This variability contrasts with the published 2012 guidelines, which recommend a duration of ACTH of 5 to 7 days for femoral artery thrombosis [36]. Similarly, wide variation was observed in the timing of catheter removal, either with or without prior administration of anticoagulant therapy (ACT). This practice also differs from the 2012 guidelines, which recommend immediate removal of the arterial catheter following imaging confirmation of arterial thrombosis, while local infusion of recombinant tissue plasminogen activator (rt-PA) through the catheter may be considered [36]. This apparent discrepancy between earlier recommendations advocating delayed catheter removal after initiation of anticoagulation and the more recent ASH recommendations reflects the absence of comparative studies rather than conflicting biological principles. Delayed removal may theoretically reduce embolization in selected high-risk patients, whereas early removal eliminates the ongoing thrombogenic stimulus. Management should be individualized according to catheter necessity, thrombus characteristics, embolic risk, and availability of alternative vascular access. Despite the broad consensus in the literature that CVC represent the most important modifiable risk factor for neonatal thromboembolic disease, substantial therapeutic uncertainties remain. Current clinical practice guidelines are based predominantly on low-quality observational evidence and expert opinion, which accounts for the considerable heterogeneity observed in routine clinical practice regarding the timing of catheter removal, the duration of anticoagulant treatment, and the management of asymptomatic thrombosis. The development of prospective multicenter studies is essential to harmonize therapeutic strategies and improve clinical outcomes.

## 3. Perinatal Stroke

Perinatal stroke is a focal vascular injury of the developing brain that happens prenatally or perinatally and includes ischemic or haemorrhagic events that involve arteries or veins, occurring from intrauterine life (most commonly from 28 weeks of gestation) through the first 28 days of life. The main subtypes include perinatal AIS, CSVT, and neonatal haemorrhagic stroke [49,50]. In some cases, perinatal stroke is defined as an event occurring between 28 weeks of gestation and the first 7 days of life, although lesions that occurred before 28 weeks’ gestation were also described [49]. Other authors expand this timeframe from 20 weeks of gestation to 28 days after birth [51]. Approximately 80% of perinatal strokes are ischemic, while the remainder is caused by CSVT or haemorrhage [49].

Diagnosis requires high-resolution imaging, and MRI diffusion-weighted imaging (DWI) is the method of choice for early detection of ischemic injury and cerebral oedema. MRA is indicated in cases of complicated delivery or a history of medical interventions, in order to depict accurately vascular lesions [49]. Assessment of the haemostatic profile and thrombophilia testing in these neonates are of limited clinical help, given that levels of protein C, protein S, antithrombin (AT), and factor XI are physiologically reduced to approximately 30% of adult levels and reach adult levels at different time points during childhood. Therefore, investigation of these factors during the neonatal period may be misleading, with limited clinical significance in the acute phase, and requires repeat testing for diagnostic confirmation. The American Stroke Association emphasizes that the diagnosis of inherited thrombophilia should be made by repeat testing at an older age, when coagulation factor levels approach adult reference ranges. Furthermore, genetic testing for mutations like Factor V leiden (FVL) and PTG mutation G20210A (PT G20210A) can be performed to identify hereditary predisposition [49]. The role of inherited and acquired thrombophilia in the pathogenesis of perinatal stroke remains controversial and has not been fully elucidated [49,52]. Due to the limited number of patients that are followed up, the effect of thrombophilia on the course of disease or risk of recurrence cannot be clearly defined [53,54]. Despite a possible association between thrombophilic risk factors and perinatal stroke, their causative role in the pathogenesis requires further systematic investigation.

### 3.1. Arterial Ischemic Stroke

The incidence of neonatal AIS is 1:4000 live births [55]. Hypoxic–ischemic stroke represents the most common cause of cerebral palsy in neonates [56]. In neonates with AIS, the most frequent clinical presentation is focal seizures, typically limited to a single limb [57,58]. Presumed Perinatal Ischemic Stroke (PPIS) refers to a child with a normal perinatal neurological history but with neurological deficits attributable to a remote focal infarction, which in turn is identified on neuroimaging later during childhood [56,59]. Neurological manifestations mainly include asymmetric motor impairment or unilateral hypertonia, seizures, or more rarely delays in cognitive and language development, as well as behavioural disorders [57,60]. Recent evidence from a systematic review and meta-analysis confirms the multifactorial origin of neonatal AIS, involving maternal, intrapartum, placental, and neonatal factors. Significant maternal associations included gestational hypertension (OR 1.90, 95% CI 1.47–2.45), gestational diabetes mellitus (OR 5.51, 95% CI 2.71–11.20), a history of infertility (OR 2.44, 95% CI 1.08–5.53), and placenta previa (OR 3.92, 95% CI 1.27–12.10). Important perinatal factors included chorioamnionitis (OR 4.35, 95% CI 2.45–7.74), fetal distress (OR 4.23, 95% CI 3.15–5.66), abnormal cardiotocography (OR 7.59, 95% CI 4.58–12.58), prolonged rupture of membranes (OR 3.02, 95% CI 1.36–6.73), resuscitation at birth (OR 4.14, 95% CI 1.59–10.76), a 5 min low Apgar score (OR 8.56, 95% CI 3.82–19.17), and umbilical arterial pH < 7.10 (OR 7.71, 95% CI 2.68–22.21). Hypoglycemia (OR 6.78, 95% CI 3.03–15.16), hypoxia (OR 7.45, 95% CI 1.09–50.80), and male sex (OR 1.51, 95% CI 1.26–1.81) were also associated with increased risk. The presence of several of these factors, particularly when accompanied by seizures, altered consciousness, abnormal tone, or feeding difficulties, should increase clinical suspicion and prompt early brain MRI with diffusion-weighted imaging [61]. The majority of lesions are in the left hemisphere, mostly within the distribution of the middle cerebral artery [57].

Supportive therapeutic measures in the management of neonatal AIS include seizure control, optimization of oxygenation, and correction of dehydration and anaemia [49,62]. Antiplatelet therapy, such as aspirin, and ACTH with low molecular weight heparin (LMWH) or unfractionated heparin (UFH) are rarely indicated, because of the low risk of AIS recurrence after a neonatal event [49]. These therapies need to be considered in exceptional cases where neonates are at high risk of recurrence, such as those with documented thrombophilia or complex congenital heart disease (excluding patent foramen ovale) [22,63].

Hyperacute therapies, such as thrombolytic interventions and mechanical thrombectomy, are rarely employed in neonates with AIS, as there is insufficient scientific evidence to support their safety and efficacy in this population [49,64]. Although micro-endovascular interventions are occasionally used in older children with arterial occlusion, the small calibre of neonatal arteries renders the application of the currently available endovascular devices impractical in this age group [62].

For infants with recurrent AIS, treatment with anticoagulation or aspirin (1–5 mg/kg/day) is recommended, while the optimal duration of anticoagulation or aspirin therapy has not been definitively specified and established.

### 3.2. Cerebral Sinovenous Thrombosis

Cerebral Sinovenous Thrombosis (CSVT) is a rare but frequently underdiagnosed condition, with mortality rates reaching 10% and neurological complications reported in up to 40% of survivors [65]. The incidence of neonatal CSVT reaches 2.6 to 12 per 100,000 live births, with a slight male predominance [66,67]. Clinical symptoms are often nonspecific, which frequently delays diagnosis and the initiation of therapy. Clinical manifestations include lethargy, irritability, or seizures, apnoea (58%), generalized neurological findings (76%), focal neurological signs (42%), coma (30%), and motor disturbances (21%) [65,68]. Less commonly, anaemia or thrombocytopenia may occur [49]. In severe cases, loss of limb function may be present [49,69].

Important clinical findings in CSVT include papilledema and other signs of increased intracranial pressure [49]. The vascular structures most involved are the superior sagittal sinus and the transverse sinuses, while in up to 30% of cases, venous infarction with subsequent haemorrhage has been reported [70]. Intraventricular haemorrhage (especially in term neonates), as well as haemorrhages involving the caudate nucleus and the thalamus, are associated with deep venous thrombosis. Subsequently, the presence of intraventricular or thalamic haemorrhage in a term or late preterm neonate dictates evaluation for CSVT [49].

Hypoxic–ischemic encephalopathy, complicated pregnancy or delivery, dehydration, prematurity, congenital heart disease, and sepsis are some of the risk factors for the development of thrombosis [71]. CSVT is also associated with therapeutic hypothermia, as a recent study reported an incidence of neonatal CSVT of 27% following therapeutic hypothermia [72]. Neonatal CSVT is likewise a multifactorial disorder, although the evidence supporting individual risk factors remains limited because of its rarity and the paucity of large observational studies [61]. Current evidence suggests that CSVT results from the interaction of maternal, perinatal, and neonatal factors. Reported maternal and perinatal contributors include preeclampsia, gestational hypertension, gestational diabetes mellitus, primiparity, twin or multiple pregnancies, chorioamnionitis, fetal distress, emergency cesarean section, complicated delivery, and perinatal asphyxia. Neonatal risk factors include prematurity, male sex, sepsis, meningitis, dehydration, heart failure, hypoxia, prolonged arterial or venous catheterization, and inherited thrombophilia [61]. Prothrombotic abnormalities may further increase susceptibility; pooled pediatric data demonstrated significant associations between cerebral venous thrombosis and the prothrombin G20210A mutation (OR 3.1, 95% CI 1.4–6.8) and FVL (OR 3.1, 95% CI 1.8–5.5), although their specific contribution to neonatal CSVT remains uncertain [73,74]. Importantly, the coexistence of multiple maternal, perinatal, and neonatal risk factors appears to substantially increase thrombotic susceptibility, underscoring the need for heightened clinical vigilance in neonates presenting with nonspecific neurological manifestations. Computed tomography (CT) or MRI, and angiography, are the preferred modalities for diagnosis, assessment, and follow-up. Repeat imaging within 5 to 7 days should be considered when deciding against anticoagulant therapy, to rule out the possibility that the thrombus is extending. Cerebral Doppler ultrasonography may also be used for initial diagnosis [49].

The ASH/ISTH guideline committee recommends anticoagulant treatment over no anticoagulant treatment in children and neonates with CSVT, with or without haemorrhage secondary to venous infarction, making a weak recommendation due to low certainty of the available evidence [37]. Management should be individualized in cases where intracranial haemorrhage is present at the time of diagnosis. It is particularly important to distinguish haemorrhage caused by venous congestion from haemorrhage of another aetiology before deciding on anticoagulant therapy. In cases of intracranial haemorrhage or other manifestations attributed to venous congestion or venous infarction, anticoagulant treatment is recommended, as there is a risk of complications from thrombus progression [37]. However, the committee acknowledges that different patient populations, such as neonates, may present variable risks of haemorrhage and neurological outcomes, which must be considered when deciding on anticoagulant therapy [37]. There are no randomised clinical trials that assessed the safety and efficacy of anticoagulant treatment in neonatal CSVT. A systematic review and meta-analysis demonstrated that anticoagulant treatment reduces the risk of thrombus extension, though it does not significantly affect the mortality or the progression of pre-existing intracranial haemorrhage [75]. Despite these encouraging findings, the absence of randomised controlled trials creates significant scientific uncertainty regarding the actual clinical benefit of therapy. There is an urgent need for well-designed, randomised, placebo-controlled trials to clearly define the role, efficacy, and safety of anticoagulant treatment in neonatal CSVT. To date, there is no evidence favouring the use of thrombolysis or thrombectomy in neonatal CSVT. In terms of treatment duration, an initial single-centre study from Toronto demonstrated that recanalization of the venous sinuses in neonates with CSVT occurs rapidly, 50% between 6 weeks and 3 months, 65% at 6 months, and 75% at 1 year. Without anticoagulant therapy, thrombus extension was observed in 10 out of 40 neonates (25%) within the first 7 days, versus only 1 out of 28 neonates who received anticoagulant treatment (*p* = 0.04) [76]. Although thrombus extension was asymptomatic in most cases, in one neonate it was associated with a new infarction. Additional data from the same research group (2011) confirmed that nearly 90% of neonates achieved complete or almost complete recanalization within 3 months of diagnosis [71]. Similarly, in a Dutch cohort study (*n* = 24), MRI at 3 months demonstrated recanalization in all surviving neonates [77], while in a European multicentre study no recurrences were reported during a 36-month follow-up period [78]. The previous observations led to the following recommendations: in neonates, provided there are no contraindications, anticoagulant treatment may be considered on an individual basis during the acute phase of CSVT, with a treatment duration of 6–12 weeks, depending on the clinical course and imaging findings [79]. Follow-up includes repeat imaging (MRI/MRV) at 3–6 months to assess recanalization and thrombus stability [80]. Overall, the available evidence consistently highlights two key findings. First, serial neuroimaging follow-up constitutes an integral component of the management of neonatal CSVT, as it enables documentation of venous sinus recanalization, assessment of thrombus stability, and contributes to determining the optimal duration of anticoagulant treatment. Second, recanalization is achieved in the majority of neonates within the first months following diagnosis, while anticoagulant treatment appears to limit thrombus propagation without significantly increasing the risk of progression of intracranial hemorrhage. Nevertheless, these conclusions are derived almost exclusively from small observational studies and retrospective case series, precluding definitive conclusions regarding which neonates derive the greatest benefit from anticoagulant treatment, the optimal duration of treatment, and whether radiological recanalization is associated with improved long-term neurodevelopmental outcomes. Consequently, the lack of neonatal-specific randomized controlled trials remains the principal barrier to the development of robust, evidence-based therapeutic recommendations.

## 4. Renal Vein Thrombosis

RVT is the commonest type of idiopathic venous thrombosis in the neonatal period, accounting for approximately 10% of all neonatal thrombotic events, and constitutes the most frequent site of thrombosis not associated with a CVC in this age group [7]. The incidence of RVT is estimated at 2.2 per 10,000 live births. Life-threatening bilateral RVT is present in approximately 30–57% of cases, and nearly 50% of these cases, the thrombus extends into the inferior vena cava (IVC) [81,82]. Risk factors include maternal diabetes mellitus, perinatal asphyxia, intrauterine growth restriction (IUGR), sepsis, and polycythaemia–hyperviscosity states [83,84]. The classic triad of a palpable abdominal mass, haematuria, and thrombocytopenia is not always present in neonates and is only found in approximately 22% of cases [82,85]. For this reason, diagnosis requires a high suspicion index when even one of these clinical features is present. The majority of neonates develop RVT within the first 72 h after birth (67%), with 26% presenting later and 7% occurring antenatally [82]. In severe cases, renal injury may develop, leading to secondary hypertension and contributing to long-term morbidity. When there is extension into the IVC, cold, cyanotic, and oedematous lower extremities may be observed. Thrombocytopenia is a common laboratory finding, and neonates presenting with isolated thrombocytopenia should be assessed for possible RVT [86]. Diagnosis can be readily established using abdominal Doppler ultrasonography. The management of neonates with RVT continues to evolve [86]. The ASH/ISTH Guideline Committee recommends the use of anticoagulant treatment in neonates with non-life-threatening RVT (cases of unilateral RVT) over no anticoagulation, to prevent hypertension, chronic kidney disease, and renal failure. This recommendation was based on observational studies of neonates with RVT, with high risk of bias and very small sample sizes [43,87]. The results showed that anticoagulant treatment can reduce the risk of chronic kidney disease and promote thrombus resolution, although differences in long-term outcomes between anticoagulant and thrombolytic therapy were small. The risk of bleeding was higher with thrombolytic therapy. Thus, most experts agree that unilateral RVT, or unilateral RVT with extension into the IVC, should be treated with anticoagulant treatment for 6–12 weeks. In contrast, for bilateral RVT or RVT associated with renal dysfunction or failure, initial thrombolytic therapy followed by anticoagulant treatment is the suggested regime [36]. RVT is associated with long-term complications, including reduced renal function and the development of systemic hypertension [88]. Although anticoagulation is widely accepted for unilateral RVT with extension into the inferior vena cava or for bilateral disease, the optimal management of isolated unilateral RVT remains less certain. This uncertainty reflects the absence of randomized neonatal studies and the observation that anticoagulation has not consistently demonstrated improvement in long-term renal outcomes. Treatment decisions should balance the theoretical benefit of preventing thrombus propagation against the increased risk of bleeding, particularly in critically ill or extremely preterm neonates.

## 5. Portal Vein Thrombosis

Neonatal PVT is estimated to occur in 3.6–36 per 1000 NICU admissions and in approximately 1:10,000 live births, and is the most common site of venous thrombosis in the neonatal period [54]. In neonates with a UVC, the incidence may reach 21–36%, depending on the duration of catheter placement and the presence of additional risk factors, such as therapeutic hypothermia [89]. The most important predisposing factor is the presence of a UVC, and thrombosis is mostly located in the left branch of the portal vein [16,20,90]. In preterm or low-birth-weight neonates undergoing routine ultrasonographic screening, the incidence of asymptomatic thrombosis of the left portal vein may reach up to 43% [90].

Risk factors for the development of PVT in neonates with a UVC include of prolonged catheter dwell time and the process of exchange transfusion through the UVC [90]. Diagnosis of PVT is established by Doppler ultrasonography. Most neonates with PVT are asymptomatic, although they may present with isolated thrombocytopenia, with or without elevated liver enzyme levels [91]. The main therapeutic goal is the prevention of portal hypertension [92]. Most thrombotic events resolve spontaneously without complications; long-term follow-up is required for the timely detection of long-term complications, such as portal hypertension [20].

In a prospective study, partial or complete thrombus resolution was observed in most patients with partial PVT (70%) after administration of anticoagulant therapy, while complete thrombus resolution occurred in only 31% of cases with complete PVT occlusion [93]. A follow-up study of neonates with PVT showed that 28% developed asymptomatic atrophy of the left hepatic lobe and 7% developed slowly progressive splenomegaly, outcomes that did not differ depending on the administration of anticoagulant therapy [16,92]. Findings from these studies emphasize an important clinical dilemma, raising the question of which subgroup of neonates merits and truly benefits from anticoagulant treatment or thrombolysis so that serious long-term complications may be prevented, including those with thrombi that have progressed or extended from the main portal vein into its branches.

Bhatt and colleagues [20] retrospectively reviewed medical records of neonates diagnosed with PVT, together with a systematic review of the literature for the evaluation of anticoagulant treatment in neonatal PVT, from 2000 to 2018. They reported that most cases of neonatal PVT are asymptomatic and follow a self-limiting course, with high rates of complete or partial thrombus resolution irrespective of anticoagulant treatment. Recently, Solgun et al. [94] reported a lack of evidence that anticoagulant treatment diminishes either short- or long-term complications. Clinically significant long-term consequences of neonatal PVT include hepatic lobe atrophy, portal hypertension, oesophageal varices, and development of collateral venous circulation. Although these complications are uncommon, long-term follow-up is essential to assess the need for early intervention and to define the exact incidence of long-term outcomes, like portal hypertension [20,94].

According to the ASH/ISTH guidelines [37] and data from 4 recent observational studies including neonates with PVT [20,94,95,96], uncertainty regarding the potential benefit or harm of anticoagulant treatment in neonates remains significant due to the limited available data. Current evidence supports the use of anticoagulation in occlusive PVT in children of all ages, whereas anticoagulant treatment in neonates with non-occlusive PVT is not recommended, and their management is limited to regular imaging follow-up for monitoring of thrombus progression. Similarly, in cases of chronic thrombosis, where portal hypertension is associated with an increased risk of oesophageal variceal bleeding, anticoagulation is contraindicated. The limited available evidence impedes the identification of neonates at high risk for complications, while potential effects of anticoagulant treatment on other clinical outcomes cannot be excluded, but there are no sufficient published data to support such associations.

The recent ASH/ISTH guidelines for the management of venous thromboembolism in children and neonates are based on limited high-quality evidence, with most recommendations stemming from observational studies and extrapolation from adult data [35,37]. For neonates with symptomatic deep vein thrombosis (DVT) or PE, anticoagulant treatment is recommended over no treatment, despite the very low certainty of evidence [47]. Data from prospective multicentre studies, such as NEOCLOT, indicate that anticoagulation reduces the risk of thrombus extension without a clear impact on mortality or thrombus resolution, and the risk of major haemorrhage may reach up to 6% [95]. However, in specific clinical scenarios, such as CVC-associated thrombosis or after trauma, anticoagulant treatment may not offer substantial benefit or may increase the risk of bleeding [97,98].

For neonates with asymptomatic thrombosis (clinically unsuspected VTE) the committee recommends either administration or withholding anticoagulant treatment, as available data from small series (<100 cases) do not show differences in thrombus progression or recurrence, and in some cases, treatment was associated with an increased risk of bleeding complications (up to 33%) [95,99,100]. Accordingly, the therapeutic decision should be individualised, taking into consideration the risk of bleeding and the severity of the underlying condition [37].

In cases of provoked VTE occurring in the presence of identifiable risk factors, the committee suggests a shorter duration of anticoagulant therapy, 6 weeks rather than 3 months, for selected low-risk patients, based on the results of the multicentre Kids-DOTT study [37,101,102]. Although neonates represented a small percentage of the study population, the trial demonstrated non-inferiority of the shorter regime in terms of thrombus recurrence and bleeding, supporting the possibility of limiting treatment duration in selected cases [103]. For neonates with unprovoked thrombosis, a duration of anticoagulant treatment of 6 to 12 months is recommended, rather than indefinite treatment [37]. Although adults with unprovoked VTE seem to benefit from extended anticoagulation, lack of paediatric data together with the impact of long-term therapy on quality of life and the bleeding risk makes indefinite treatment less acceptable in younger patients [104,105]. Given the risk of delayed portal hypertension, long-term follow-up for at least five years has been suggested to facilitate early detection of complications and reduce the likelihood of gastrointestinal hemorrhage during childhood [91]. This apparent discrepancy between earlier recommendations advocating delayed catheter removal after initiation of anticoagulation and the more recent ASH recommendations reflects the absence of comparative studies rather than conflicting biological principles. Delayed removal may theoretically reduce embolization in selected high-risk patients, whereas early removal eliminates the ongoing thrombogenic stimulus. Therefore, management should be individualized according to catheter necessity, thrombus characteristics, embolic risk, and availability of alternative vascular access [91]. In clinical practice, this decision should be guided by thrombus characteristics (occlusive versus non-occlusive disease, thrombus extent and progression on serial Doppler ultrasonography), evidence of portal hypertension or hepatic dysfunction, the presence of ongoing catheter-related risk factors, the estimated bleeding risk, and the overall clinical condition of the neonate [37]. Overall, the available evidence indicates that neonatal PVT generally follows a benign and self-limiting course, with spontaneous or partial thrombus resolution occurring in most cases, irrespective of anticoagulant treatment. The current literature does not demonstrate a clear benefit of anticoagulant treatment in preventing long-term complications, such as portal hypertension and chronic liver injury. The most important unmet clinical need is not the routine administration of anticoagulant treatment, but rather the early identification of the small subset of neonates at increased risk of developing progressive portal hypertension or other chronic hepatic complications who may benefit from closer surveillance and individualized therapeutic management. Until high-quality, neonatal-specific evidence becomes available, long-term follow-up with Doppler ultrasonography remains the cornerstone of the clinical management of these patients.

## 6. Spontaneous Arterial Thrombosis

Spontaneous arterial thromboses are most frequently located in the aorta and may mimic congenital heart disease [52]. A reduction in pulses or the presence of cold, mottled extremities should raise suspicion for arterial thrombosis [106].

In neonates and children with right atrial thrombosis (RAT), the ASH/ISTH Guideline Committee recommends the use of anticoagulant treatment rather than no treatment for patients with high-risk features and a low estimated bleeding risk [37]. These recommendations illustrate the current shift away from routine anticoagulation for all intracardiac thrombi toward a risk-stratified approach. Since many right atrial thrombi remain clinically stable or resolve spontaneously, exposing all neonates to anticoagulation may unnecessarily increase bleeding risk, whereas patients with high-risk thrombus characteristics appear more likely to benefit from active treatment. High-risk features include large thrombus size or atypical shape, thrombus mobility, location affecting blood flow or involving the tricuspid valve, the presence of a right-to-left intracardiac shunt, presence of a CVC, or clinical symptoms like arrhythmias or haemodynamic dysfunction. The decision to start anticoagulant treatment should be individualised, carefully balancing the risk of thrombosis with the estimated risk of bleeding.

On the contrary, in patients without high-risk features or with an unacceptably high estimated bleeding risk, withholding anticoagulant treatment is recommended over treatment with anticoagulants, as available studies do not show any clinical benefit, and anticoagulation is associated with an increased risk of bleeding [37]. For neonates and children with RAT who require antithrombotic therapy, the committee recommends using only anticoagulant treatment rather than thrombolytic therapy followed by anticoagulation [37]. In most cases, anticoagulation is sufficient, while thrombolytic therapy should be considered only in selected cases with a large or mobile thrombus and haemodynamic compromise, considering feasibility, patient and family acceptance, and the risks from the intervention [36,37].

Overall, the quality of available evidence in neonatal populations remains very low, with scarce randomised controlled trials [37]. The need for future, targeted research in neonates is imperative to determine the optimal timing of initiation and duration of anticoagulant treatment, to assess the associated bleeding risk, and to investigate the impact of treatment on clinical outcomes, recurrence, and long-term complications such as post-thrombotic syndrome [52,107]. Table 2 provides a concise overview of the most recently published guidelines for the management of thromboembolic disease in neonates.

## 7. Diagnostic Approach

**Figure 2:**
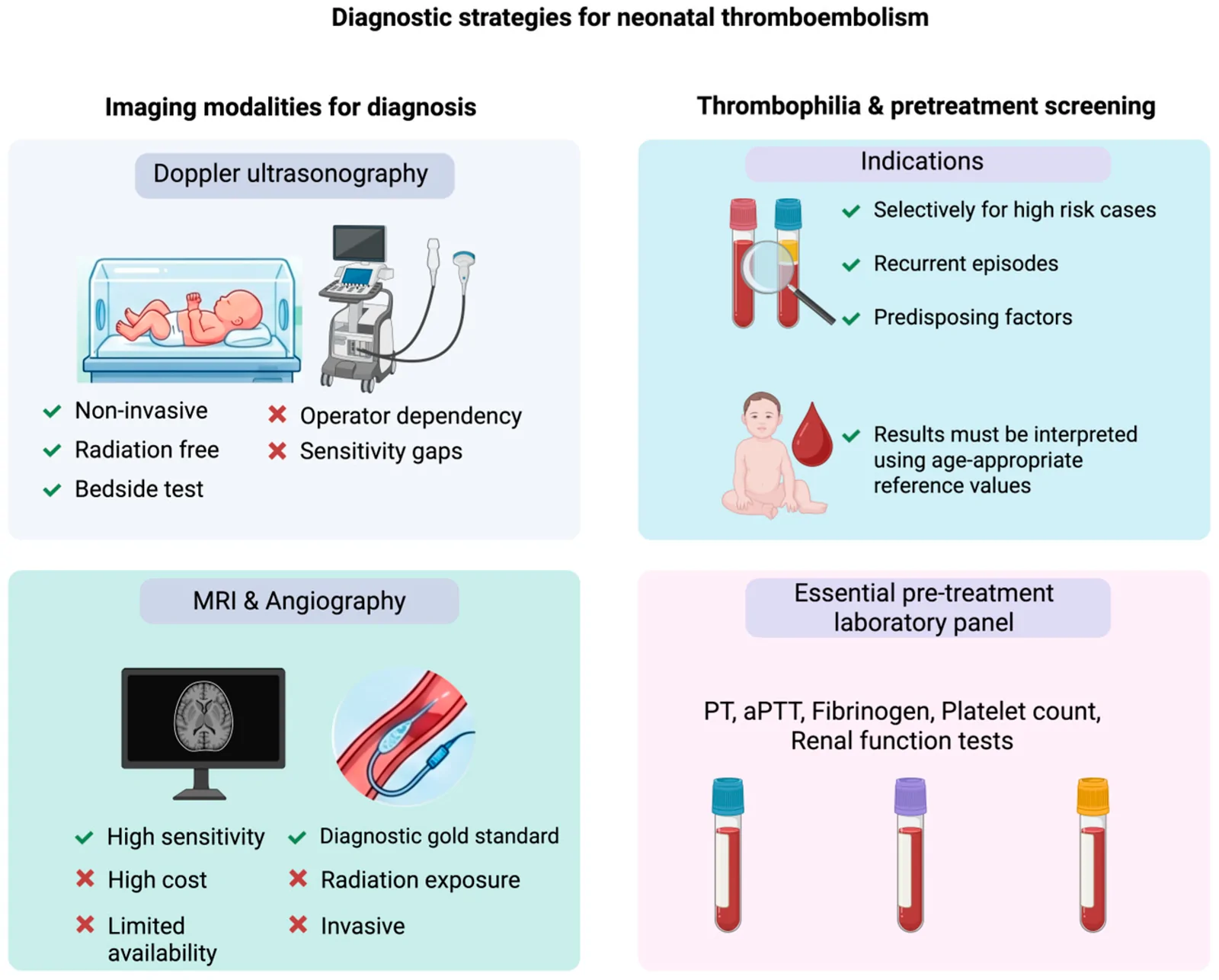
**Diagnostic strategies for neonatal thromboembolism.** Neonatal thromboembolism is primarily diagnosed using Doppler ultrasonography, a non-invasive, bedside method without radiation, though its accuracy depends on the operator and is limited in deeper vessels or challenging clinical conditions. Imaging findings vary over time and can help assess prognosis, while more invasive methods like angiography are rarely used despite being the gold standard. MRI, particularly MR venography for suspected cerebral venous thrombosis, and CT are complementary imaging modalities in selected cases when ultrasonography is inconclusive or deeper vascular territories require further evaluation. Thrombophilia screening is not routinely required, as most neonatal thrombosis is catheter-related, but may be considered in recurrent or unexplained cases. Testing must account for age-related variations, and results require cautious interpretation due to limitations and potential implications. MR: magnetic resonance, CT: computed tomography, MRI: magnetic resonance imaging, PT: prothrombin time, aPTT: activated partial thromboplastin Time. Created in BioRender. Lianou, A. (2026) https://BioRender.com/fyhlqth.

Multiple imaging modalities can be used for the diagnosis of TE in neonates. Doppler ultrasonography is the imaging modality of choice for confirmation of the diagnosis of venous or arterial thrombosis in most cases [108]. The advantages of this method include the fact that it is non-invasive, does not require exposure to ionizing radiation, and can be performed at the patient’s bedside. However, it is a significantly operator-dependent technique and demonstrates reduced sensitivity in detecting thrombi that extend into the deep veins, such as the portal vein, the inferior vena cava, the central vessels of the upper extremities (such as the subclavian and innominate veins), the superior vena cava, or the right atrium. The ultrasonographic features of thrombosis depend on the timing of the examination [109,110,111]. For example, in RVT during the first days, echogenic bands are observed in a peripheral focal area of the affected kidney. Within the first week, the kidney appears enlarged and echogenic, with visible but less echogenic medullary pyramids. With the resolution of the oedema, the kidney develops a heterogeneous texture with loss of corticomedullary differentiation, which may progress to atrophy with focal scarring, or complete recovery. Colour Doppler ultrasonography may demonstrate absence of intrarenal or renal venous flow in the early stages of RVT [109], and ultrasonographic findings can be used to prognosticate. Non-compressibility of the vessel, with or without the presence of a visualized intraluminal thrombus, confirms the diagnosis of catheter-related thrombosis. The presence of the catheter itself may affect the sensitivity of the examination, as it may reduce luminal compressibility, particularly in preterm neonates with small vessel diameters, thus impeding the accurate detection of thrombus presence [30]. In addition, preterm or critically ill neonates may have low stroke volume, which further complicates the interpretation of Doppler findings when assessing for a suspected arterial thrombosis. Furthermore, sensitivity is reduced in vessels located under areas of skin covered with dressings, or in cases with other interposing abnormalities. Angiography/venography is considered the gold standard for the diagnosis of VTE. Nevertheless, it is rarely feasible in neonates because it requires adequate peripheral venous access, frequently necessitates sedation, and exposes the patient to ionizing radiation. Thus, its use in routine neonatal clinical practice is limited [4,112]. Alternative imaging modalities, such as MRI and CT, are also available, but are rarely considered necessary. MR angiography has the advantage of being a non-invasive technique with high sensitivity and specificity for the detection of intrathoracic and intracranial thrombosis, without the need for exposure to radiation (in contrast to CT). Its use is nevertheless limited by its higher cost compared with CT and its limited availability in some centres [108,113,114]. The choice of the appropriate imaging modality depends on the anatomical location of the thrombosis as well as the clinical state of the patient (Figure 2).

Recent advances in MRI techniques have substantially expanded the capabilities for imaging assessment of neonatal thromboembolic disease. In particular, MR venography is currently the imaging modality of choice for the diagnosis of CSVT, as it enables accurate visualization of the cerebral venous system and confirmation of thrombus presence while simultaneously providing valuable information on associated intracranial abnormalities, including cerebral edema, venous ischemic infarction, and hemorrhagic transformation. Furthermore, MR venography facilitates longitudinal assessment of thrombus evolution and venous sinus recanalization during follow-up without exposing patients to ionizing radiation. Consequently, MR venography is expected to play an increasingly important role in the diagnostic evaluation of neonates with suspected CSVT [115].

### 7.1. Thrombophilia Screening

The use of thrombophilia screening in neonates with thrombosis remains uncertain. Inherited thrombophilias are associated with an increased risk of recurrence. Thrombophilia screening is not essential for the vast majority of neonates with thrombosis. Most cases of neonatal thrombosis are associated with the presence of a catheter and the diagnostic value of thrombophilia testing in this context is limited [116]. Data suggest that the coexistence of multiple thrombophilic factors or the combination of inherited thrombophilia with one or more clinical risk factors further increases the likelihood of neonatal thromboembolism [117]. Although clinical factors appear to play a more important role, investigation for inherited thrombophilia may be appropriate in selected cases, such as neonates with recurrent thrombotic episodes, cases of thrombosis without an obvious predisposing factor, a positive family history of venous thromboembolism, or based on the severity, location, and number of acquired risk factors [118]. Given that investigation of thrombophilic markers requires a large volume of blood sample, a stepwise approach is recommended, where specific tests are performed during the acute episode, while others can be carried out at the age of 3–6 months. If the decision is made to perform thrombophilia testing in a neonate, the tests performed are generally the same as those performed in older children (summarized in Table 3).

The only exception has to do with testing for antiphospholipid antibodies, which is performed in maternal blood samples rather than neonatal blood. Developmental haemostasis is characterized by age-related alterations in reference values for functional assays and for tests measuring haemostatic protein levels, depending on gestational age and postnatal age [119]. Consequently, interpretation of the results should take into consideration age-appropriate reference values. In case of an abnormal result, repeat testing is recommended after 4–6 weeks, and parents should be informed and appropriately counselled regarding the results and their potential repercussions [108]. In contrast, molecular tests, such as detection of the FVL mutation or the PTG G20210A mutation, are not affected by age and therefore are not limited in the same way. With the increasing number of publications on the use of molecular assays in deficiencies of natural anticoagulants, or in other causes of APC resistance (APCR) beyond FVL, their gradual adoption for identifying causes of thrombophilia is anticipated when conventional/phenotypic tests fail and for optimal patient stratification [108,120].

At the same time, both conventional and molecular tests have limitations, including false-positive or false-negative results, identification of variants without a clear causative association, limited availability, high cost, and potential social or insurance implications. This highlights the need for careful interpretation of test results and individualized clinical assessment, while treatment aims at achieving management of thrombophilia or thrombosis in general, rather than that of a specific genetic variant [120].

### 7.2. Supplementary Investigations

If anticoagulant treatment is planned, it is recommended to conduct a baseline preoperative laboratory assessment, and a cranial ultrasound to exclude intracranial or intraventricular haemorrhage, especially in preterm neonates. Essential pre-treatment laboratory tests include activated partial thromboplastin time (aPTT), prothrombin time (PT) and international normalised ratio (INR), fibrinogen concentration in plasma, full blood count (including platelets), renal function tests (urea and creatinine), transaminases (particularly in case of planned treatment with direct acting οral αanticoagulants, (DOACs)) [108,119].

## 8. Emerging Diagnostic and Therapeutic Perspectives

Despite the substantial progress achieved in recent years in the understanding and management of neonatal thromboembolic disease, its diagnosis and therapeutic management continue to rely on limited evidence, derived primarily from observational studies or extrapolated from data obtained in older children and adults. Developmental hemostasis, the rarity of the condition, and the heterogeneity of the neonatal population constitute major challenges to the development of reliable diagnostic and therapeutic strategies, underscoring the need for a more individualized approach. The identification of reliable biomarkers for the early recognition of neonates at high risk of thromboembolic disease represents one of the foremost contemporary research challenges. The systematic review by Pelland-Marcotte et al. [121] focused on the pediatric population, in which neonates constituted only a subgroup. The authors concluded that, although several candidate biomarkers, including D-dimers, factor VIII (FVIII), C-reactive protein (CRP), thrombin–antithrombin (TAT) complexes, and, in selected populations, albumin, demonstrated promising findings in individual studies, none currently possesses sufficient diagnostic or prognostic accuracy to warrant integration into routine clinical practice. The substantial heterogeneity among studies, differences in the measurement methodologies employed, and the high risk of bias considerably limit the generalizability of these findings. Moreover, even the most extensively investigated biomarkers, such as D-dimers and FVIII, require further prospective validation before they can be adopted for clinical use. Nevertheless, extrapolation of these conclusions to the neonatal population should be undertaken with considerable caution. Neonates constitute a unique population characterized by distinct quantitative and qualitative differences in their hemostatic system compared with older children and adults, a phenomenon referred to as developmental hemostasis, which influences both the physiological reference ranges and the interpretation of hemostatic biomarkers. Specifically, even healthy neonates physiologically exhibit higher D-dimer concentrations than adults, despite the absence of clinical or ultrasonographic evidence of thrombosis and with otherwise normal coagulation parameters. These elevated D-dimer concentrations are thought to reflect reduced renal clearance of D-dimers, as well as the physiological changes accompanying postnatal closure of the ductus venosus and ductus arteriosus. Tthe direct application of adult diagnostic cut-off values during the neonatal period is inappropriate, highlighting the necessity for the use of age-specific reference ranges [122]. In parallel, emerging global hemostatic assays, such as thromboelastography (TEG) and rotational thromboelastometry (ROTEM), provide a more comprehensive assessment of functional hemostasis than conventional coagulation tests. Although these techniques have already demonstrated particularly promising results in the evaluation of neonatal hemostasis and, especially, hemorrhagic disorders, their role in the diagnosis, risk stratification, or therapeutic guidance of neonatal thromboembolic disease remains insufficiently established [123,124,125,126,127,128,129,130].

Concurrently, advances in imaging modalities represent another important direction in the diagnostic evaluation of neonatal thromboembolic disease. Doppler ultrasonography remains the first-line imaging modality because of its widespread availability and the feasibility of bedside application in the neonatal intensive care unit (NICU). Nevertheless, its sensitivity is limited, particularly for thrombosis involving the cerebral venous sinuses and deep cerebral veins. In contrast, magnetic resonance venography (MRV) is currently regarded as the imaging modality of choice, with a reported sensitivity approaching 90%, as it enables not only accurate visualization of the thrombus but also assessment of secondary complications, including cerebral edema, ischemic or hemorrhagic infarction, as well as longitudinal monitoring of disease progression [122,131].

The recent systematic review and meta-analysis by Pelland-Marcotte et al. [132], which focused exclusively on neonates, demonstrated that the available evidence is even more limited. The authors found insufficient data to evaluate thrombosis-related biomarkers during the neonatal period, and their analysis therefore focused primarily on clinical risk factors, identifying preeclampsia, low birth weight, congenital heart disease, and neonatal infection as the most significant predictors. Furthermore, they emphasized that future research should focus on the identification of reliable biomarkers, the development of neonatal risk prediction models, and the application of advanced computational approaches, including machine learning, with the aim of enabling early identification of neonates at high risk and the implementation of individualized prevention and management strategies.

Advances in molecular diagnostics have substantially expanded the possibilities for investigating inherited thrombophilia, representing one of the most promising developments toward a more personalized diagnostic approach to neonatal thromboembolic disease [120]. Studies in the pediatric population have demonstrated that genetic abnormalities, including FVL (G1691A), the prothrombin G20210A mutation, and deficiencies of protein C, protein S, and antithrombin, are associated with an increased risk of venous thromboembolism, particularly when multiple thrombophilic abnormalities coexist [14,24,114,133,134,135,136,137,138]. The available data in neonates remain limited, and interpretation of the findings requires particular caution, as developmental hemostasis and the predominant contribution of acquired risk factors significantly modify the overall thrombotic risk. In parallel, the application of targeted gene panels and next-generation sequencing (NGS) techniques offers new perspectives for the identification of rare or complex genetic causes of thrombophilia, particularly in neonates with idiopathic or recurrent thrombosis, unusual thrombotic sites, or a strong family history. These approaches may contribute to improving recurrence risk assessment, genetic counseling, and long-term therapeutic planning. Despite these potential benefits,, given that the majority of neonatal thrombotic events are associated with acquired risk factors, such as CVC, sepsis, and congenital heart disease, routine universal molecular testing or thrombophilia screening is not currently recommended [37,120]. Instead, their use should be individualized and restricted to selected patients in whom the results are likely to have a meaningful impact on clinical management.

Beyond the identification of individual risk factors, one of the major challenges in neonatal thrombosis is the development of reliable risk stratification models capable of identifying neonates at the highest risk of thromboembolic complications before clinical presentation. Current evidence indicates that neonatal thrombosis is typically the result of multiple interacting risk factors rather than a single isolated event [24]. Sepsis and central vascular catheterization remain the predominant acquired risk factors, while prematurity, low birth weight, congenital heart disease, perinatal asphyxia, maternal disorders (including preeclampsia and diabetes), and inherited thrombophilia may further increase thrombotic susceptibility. Importantly, these factors rarely act independently but rather synergistically, reflecting the unique developmental characteristics of neonatal hemostasis [24]. Future research should therefore move beyond the evaluation of isolated biomarkers toward the development of integrated neonatal risk prediction models combining clinical characteristics, laboratory parameters, imaging findings, and genetic information. The incorporation of machine-learning algorithms and artificial intelligence may further improve individualized risk prediction, allowing earlier identification of high-risk neonates and more targeted preventive strategies.

Alongside advances in diagnostic approaches, significant progress has also been achieved in the field of anticoagulant treatment. Beyond the established anticoagulant agents, which continue to represent the first-line treatment during the neonatal period, and LMWH, which remains the preferred therapeutic option in neonates, newer anticoagulant strategies have been developed in recent years, expanding the available treatment options in the pediatric population. These emerging approaches have not yet been established as standard therapies in the neonatal period [37,49,139,140,141,142]. Vitamin K antagonists (VKAs), primarily represented by warfarin, are now rarely used in neonates, as the physiologically low levels of vitamin K-dependent coagulation factors, limited vitamin K intake, difficulties in maintaining a therapeutic INR range, interactions with dietary factors, and the lack of suitable pharmaceutical formulations substantially restrict their safety and practical applicability in this population [108,142,143,144]. Intravenous DTIs, primarily bivalirudin and argatroban, represent an important alternative therapeutic option in cases of heparin-induced thrombocytopenia (HIT) or heparin resistance, particularly in neonates undergoing Extracorporeal Membrane Oxygenation (ECMO) or cardiac surgery [145,146]. The absence of a specific antidote, the considerable pharmacokinetic variability, and the requirement for close laboratory monitoring limit their use to specialized centers [114,147]. Similarly, DOACs, primarily rivaroxaban and dabigatran, represent one of the most significant recent advances in pediatric anticoagulant treatment [148]. The EINSTEIN-Jr and DIVERSITY studies [149,150]. demonstrated comparable efficacy and safety of DOACs compared with conventional anticoagulant treatment in children with venous thromboembolism, leading to the approval of these agents for selected pediatric patients. Despite these encouraging results, available data regarding neonates remain insufficient, as preterm infants were excluded from clinical trials and the number of term neonates enrolled was extremely limited [148,151,152]. Furthermore, developmental hemostasis, immaturity of renal and hepatic function, and variable gastrointestinal absorption present significant challenges to the safe implementation of these agents during the neonatal period. Despite substantial advances in anticoagulant treatment, LMWH remains the preferred first-line treatment for neonatal thromboembolic disease. Specifically designed pharmacokinetic studies and prospective clinical trials are required before these newer agents can be incorporated into routine neonatal clinical practice [37].

The limited evidence supporting anticoagulant treatment during the neonatal period reflects the broader challenges associated with conducting clinical trials in neonates, a population that for many years has been characterized as “therapeutic orphans,” as many medications were administered without adequate scientific evidence or were used outside their approved indications [153,154]. Conducting randomized clinical trials in the neonatal population is particularly challenging, given the substantial heterogeneity of neonates with regard to gestational age, birth weight, and underlying medical conditions, while the rarity of neonatal thromboembolic disease further complicates the recruitment of adequate numbers of patients [24,155,156]. Moreover, developmental hemostasis, immaturity of hepatic and renal function, and significant age-related differences in the pharmacokinetics and pharmacodynamics of anticoagulant agents preclude the direct extrapolation of findings from studies conducted in adults or older children. Prolonged hospitalization in the NICU, the need for specialized imaging investigations, anticoagulant treatment, and long-term follow-up for potential chronic complications substantially increase healthcare resource utilization [157]. Furthermore, long-term neurological, renal, or hepatic complications may require multidisciplinary follow-up and therapeutic interventions throughout childhood [4,158]. In parallel, significant practical and ethical challenges, such as the requirement for obtaining parental informed consent in urgent clinical situations, further limit the feasibility of designing and conducting adequately powered clinical trials [159]. Consequently, most therapeutic recommendations for neonatal thromboembolic disease continue to rely primarily on observational studies, expert opinion, and extrapolation from larger pediatric populations. The development of international multicenter neonatal networks and specifically designed prospective clinical trials represents an essential prerequisite for establishing stronger, neonate-specific therapeutic guidelines. Beyond its immediate clinical consequences, neonatal thromboembolic disease is also associated with a substantial burden on healthcare systems. Yet, available data regarding the true economic impact of neonatal thromboembolic disease remain limited. Future studies evaluating both clinical and economic outcomes may contribute to optimizing resource allocation and to the development of more effective prevention and management strategies. As a narrative review, this article provides a synthesis of the currently available epidemiological data, neonatal evidence, and international guideline recommendations. 

## 9. Conclusions and Future Directions

Neonatal thromboembolic disease represents a multifactorial and evolving clinical challenge, shaped by the interplay between developmental hemostasis, increasing survival of high-risk neonates, and the widespread use of invasive supportive technologies. Despite advances in neonatal care and diagnostic capabilities, significant uncertainties persist regarding optimal diagnostic and therapeutic strategies. Clinical decision-making remains particularly challenging because of the unique developmental characteristics of neonatal hemostasis, the heterogeneity of clinical presentations, and the limited availability of high-quality evidence, highlighting the need for a nuanced and individualized approach to diagnosis and management.

Current management strategies are largely guided by expert consensus and extrapolation from data in older populations, resulting in significant variability in clinical practice. Anticoagulant therapy remains the cornerstone of treatment in many scenarios, yet its indications, timing, and duration are not well defined, particularly in asymptomatic or low-risk cases. Similarly, the role of thrombolytic therapy is limited and reserved for selected high-risk situations. Long-term outcomes of neonatal thrombosis remain insufficiently characterized. Complications such as post-thrombotic syndrome, chronic kidney disease, portal hypertension, and neurodevelopmental impairment may emerge months or years after the initial event. The lack of standardized follow-up protocols further limits our understanding of the true burden of disease.

In conclusion, advancing the care of neonates with thromboembolic disease will depend on bridging current evidence gaps through rigorous research, improving preventive strategies, and adopting individualized, risk-based approaches that balance efficacy with safety.

*Future research efforts should focus on several key priorities:*

- Standardization of diagnostic pathways, including the role of advanced imaging and biomarkers
- Development of neonatal-specific risk stratification models to guide therapeutic decision-making
- Optimization of catheter-related practices to reduce the incidence of preventable thrombosis
- Long-term outcome studies to better define the burden of chronic complications and inform follow-up strategies
- Refinement of thrombophilia testing, including the integration of molecular diagnostics and age-appropriate reference standards
- Prospective, multicenter randomized controlled trials to evaluate the safety and efficacy of anticoagulant and thrombolytic therapies in neonates
- Collaborative international registries and networks to overcome the limitations of small sample sizes and to generate high-quality data in this vulnerable population.

## Figures and Tables

**Table 2:** A concise overview of the most recently published guidelines for the management of thromboembolic disease in neonates.

Type of Thrombosis	Clinical Category/Severity	Management	Strength of Recommendation
**DVT/PE**	Symptomatic	ACTRover no treatment	Conditional recommendation, based on very low certainty of evidence regarding outcomes [37]
	Clinically non-suspicious (previously called asymptomatic)	Individualized decision: ACTR or monitoring	
	Provoked	Duration of ATC ≤ 3 months	
	Unprovoked	ATC for 6–12 months	
**CVC-associated thrombosis**	Functioning catheter whose presence is essential	Maintaining the CVC with simultaneous administration of ACTR	Conditional recommendation, based on very low certainty of evidence regarding outcomes [37]
	Symptomatic thrombosis associated with CVC without any further need for venous access or with a non-functioning CVC	Instant or delayed removal	
	Deterioration despite ACTR	Individualized decision to maintain or remove catheter	
**RVT**	Unilateral RVT without renal dysfunction or extension to the IVC	Initiation of ACTR	Strong recommendation, based on very low certainty of evidence regarding outcomes [37]
	Unilateral RVT with extension to the IVC without renal dysfunction	ACTR	
	Bilateral RVT or Unilateral RVT with renal dysfunction/renal failure	Thrombolytic therapy rt-TPA, followed by ACTR	Conditional recommendation, based on very low certainty of evidence regarding outcomes [37]
**RAT**	With risk fors	ACTR over no treatment avoid thrombolysis or surgical thrombectomy (only in haemodynamic instability or large mobile thrombus)	Conditional recommendation, based on very low certainty of evidence [37]
	Without risk fors	No ACTR	
**PVT**	Occlusive/Following transplantation/Idiopathic	ACTR	Conditional recommendation, based on very low certainty of evidence regarding outcomes [37]
	Non-occlusive PVT also for children having already developed portal hypertension secondary to PVT	Monitoring without ATC	
**CSVT**	Without haemorrhage	ACTR	Conditional recommendation, based on very low certainty of evidence derived from paediatric data [37]
	With haemorrhage	Individualized ACTR with caution	
	Large thrombotic extension	No routine thrombolysis- only ACTR	
**Purpura fulminans PF**	Chronic phase	Protein C replacement therapy over ACTR	Conditional recommendation, based on very low certainty of evidence derived from paediatric data [37]
	Acute phase	Combination of ACTR + Protein C	
**Arterial Thrombosis** [36,37]			
**Associated with femoral arterial catheter**	Not limb-threatening	ACTR	—
	Limb or organ threatening	ACTR initiation- if no response within 24–48 h, transition to thrombolytic therapy	—
**Associated with peripheral arterial line**	Not threatening	ACTR	—
		Thrombolytic therapy followed by ACTR	—
**Spontaneous or associated with UAC**	Not threatening	ACTR	—
	Limb or organ threatening	Thrombolytic therapy followed by ACTR	—

DVT: Deep Vein Thrombosis, ACTR: Anticoagulant Therapy, CVC: Central Venous Catheter, CSVT: Cerebral Sinovenous Thrombosis, IVC: Inferior Vena Cava, PE: Pulmonary Embolism, PF: Purpura Fulminans, PVT: Portal Vein Thrombosis, RAT: Right Atrium Thrombosis, rt-TPA/TPA: recombinant tissue Plasminogen Activator/tissue Plasminogen Activator, RVT: Renal Vein Thrombosis, UAC: Umbilical Arterial Catheter.

**Table 3:** Diagnostic stepwise approach of neonatal thrombophilia.

Laboratory Investigations	If Other Acquired Risk Factors ARE Present	If No Other Acquired Risk Factors Are Present
Antiphospholipid Antibodies (IgG, IgM), anticardiolipin, lupus anticoagulant	☑	☑
Protein C, activity	☑	☑
Protein S, activity	☑	☑
Lipoprotein(a)	☑	☑
Plasminogen (If thrombolytic therapy is considered)	☑	☑
Antithrombin (AT), activity	☑	☑
Genetic test for Factor V Leiden mutation	☑	☑
Genetic test for factor II (prothrombin) G20210A mutation	☑	☑
Polymorphism of Plasminogen Activator Inhibitor (PAI-1) 4G/5G	–	☑
Homocysteine (if increased, test mother for Methylenetetrahydrofolate Reductase (MTHFR) mutation)	–	☑
FVIII, activity	–	☑
FXII, activity	–	☑
Plasminogen, activity	–	☑
Heparin Cofactor (HCII)	–	☑

## Data Availability

Data are contained within the article.
